# The role of bioethics services in paediatric intensive care units: a qualitative descriptive study

**DOI:** 10.1186/s12910-024-01017-z

**Published:** 2024-02-19

**Authors:** Denise Alexander, Mary Quirke, Jo Greene, Lorna Cassidy, Carol Hilliard, Maria Brenner

**Affiliations:** 1 School of Nursing, Midwifery and Health Systems, University College Dublin, Dublin, Ireland; 2 Maynooth University, Maynooth, Ireland; 3 Children’s Health Ireland at Crumlin, Dublin, Ireland

**Keywords:** Child, Clinical bioethics, Functionality, Technology dependence, Invasive long-term ventilation, Innovation

## Abstract

**Background:**

There is considerable variation in the functionality of bioethical services in different institutions and countries for children in hospital, despite new challenges due to increasing technology supports for children with serious illness and medical complexity. We aimed to understand how bioethics services address bioethical concerns that are increasingly encountered in paediatric intensive care.

**Methods:**

A qualitative descriptive design was used to describe clinician’s perspectives on the functionality of clinical bioethics services for paediatric intensive care units. Clinicians who were members of formal or informal clinical bioethics groups, or who were closely involved with the process of working through ethically challenging decisions, were interviewed. Interviews took place online. Resulting transcripts were analysed using thematic analysis.

**Results:**

From 33 interviews, we identified four themes that described the functionality of bioethics services when a child requires technology to sustain life: striving for consensus; the importance of guidelines; a structure that facilitates a time-sensitive and relevant response; and strong leadership and teamwork.

**Conclusions:**

Clinical bioethics services have the potential to expand their role due to the challenges brought by advancing medical technology and the increasing options it brings for treatment. Further work is needed to identify where and how bioethics services can evolve and adapt to fully address the needs of the decision-makers in PICU.

**Supplementary Information:**

The online version contains supplementary material available at 10.1186/s12910-024-01017-z.

## Introduction

Clinical bioethics services exist to help identify and clarify the ethical aspects of medical decisions [1–6]. The development of increasingly sophisticated life-sustaining technology has arguably altered the goals of care, in that a cohort of children are now able to survive diseases that relatively recently would have proved fatal. For example, clinicians often find that technology such as invasive long-term ventilation (I-LTV), is initiated for children whose condition is unlikely to improve [7–9]. Deep discussions leading up to this important juncture of care is imperative, given the consequences of the initiation of technology dependence for many children and young people [10]. Clinical bioethics services have the potential to expand their role because of the challenges brought by advancing medical technology and the increasing options it brings for treatments [11]. However, currently there is considerable variation in the functionality of bioethical services across different institutions and countries [12–14]. Through the perspectives of the clinicians who contribute to bioethics discussions, we aimed to provide a greater understanding of the current functionality of clinical bioethics services at a time when a child requires life sustaining technology, using I-LTV as an exemplar. This process allows us to better understand how bioethics services may be able to address some of the bioethical concerns that are increasingly found in modern paediatric intensive care units (PICUs). We explored this potential through the concept of Rogers’ Theory of Diffusion of Innovation [15]. This paper is part of a programme of research conducted within the TechChild project, funded by the European Research Council (ERC).

## Methods

A qualitative descriptive design was chosen to describe clinician’s perspectives on the contemporary functionality of clinical bioethics services for paediatric intensive care units [16]. We were guided by Guba and Lincoln [17] in ensuring methodological and analytical rigour, by seeking credibility, transferability, dependability and confirmability in all aspects of this research. This informed rigour in recruitment, data collection, and data analysis.

### Recruitment

The target population was members of formal or informal clinical bioethics groups, or professionals closely involved with the process of working through ethically challenging decisions, as part of their clinical work. We recruited our participants online using purposive sampling in three ways. 1). Through the European Children’s Hospital Organization (ECHO); members of ECHO were invited via a gatekeeper to contact a researcher if they were interested in participating. 2). Individuals who previously engaged with the project as participants in separate phases, and expressed willingness to contribute further to the wider TechChild project, were invited, via email, to take part. 3). Those who chose to participate were encouraged to snowball the invitations to colleagues. On receipt of an email from interested participants, the researcher emailed a flyer about the project and its aims, a participant information leaflet (PIL), and a link to an online consent form. Subsequently, the researchers contacted the participants to arrange a date and time for the online interview.

### Data collection

We conducted semi-structured individual interviews online via Zoom [18] at a time of convenience for the participant. To ensure privacy, participants and researchers were the only individuals present at the time of interview. The main body of the interview began with a small number of demographic questions, and confirmation that the consent form had been understood and completed. We then asked one main question (supplementary file 1):

Can you tell me about your experience of working with clinicians on the bioethics of initiating invasive long-term ventilation to sustain a child’s life? Are there any cases that particularly resonate with you?

The participant led the interview, and the researcher prompted for more information and explored points of interest during the conversation. Most interviews lasted between 30 and 60 min and recordings were stored on a password protected institution owned computer. Immediately after each interview, the audio file was transferred via virtual private network (VPN) to a secure encrypted server in the host institution. Files were pseudo-anonymised to protect the participants’ identity. The video files and the copies of the audio files were deleted from the researcher’s computer following upload. We recognised that the subject matter of the interviews was sensitive and might be distressing for participants as they recalled certain events or patient care episodes. A distress protocol was developed in which the interviewer would pause, cancel, or defer the interview following consultation with the participant. The participants were also advised in relation to accessing supports through their own organisational structures. After each interview, the interviewer completed a reflective observation sheet and debriefed with another researcher in the team where necessary. This peer-support was a particular strength in our research group as the areas of discussion in interviews were often concerning very sensitive issues. The project team held twice-weekly meetings in order to discuss the interviews, in terms of issues that were arising of particular significance.

### Data analysis

Data analysis was guided by the techniques of thematic analysis, as described by Braun and Clarke [19]. This involved the researchers familiarising themselves with the data by listening to the interview recordings and reading and re-reading the transcriptions. Initial open codes were generated as a result. The initial themes were reviewed extensively and discussed; data was gathered until thematic saturation was achieved.

## Results

Thirty-three interviews were completed between December 2021 and May 2022 (Table 1). Twenty-three participants were physicians, five were nurses, and five were allied healthcare professionals. Sixteen participants were female and 17 participants were male. The majority of the participants were European (*n* = 18) or from the United States (*n* = 11); the remaining participants were based in Central and South America, Africa, Asia, or Australasia. Only ten participants stated they were on a formal ethics committee, a further 17 were not part of a formal ethics service, and either made decisions themselves, informally with colleagues, or were part of bioethics discussion groups they had established with other clinicians to discuss ethical issues. These groups often met regularly, such as once a month. Six participants had no access to ethical support, and dealt with issues alone, or had no opportunity to voice any ethical concerns.

**Table 1:** Participant profile

Profile variable	
**Role**	
Physician	23
Nurse	5
Allied Health Professional*	5
**Gender**	
Female	16
Male	17
**Location**	
North America	11
Europe	18
South America	1
Australasia	1
Asia	1
Africa	1
**Access to bioethics support**	
Member of bioethics committee	10 (8 Physicians, 2 Allied Health Professionals)
Not a member of a bioethics committee, has access to a bioethics discussion group	17
No access to ethical support	6

*Includes Chaplain, Physiotherapist, Pulmonologist, Respiratory therapist

One of the main topics of the interviews was the functioning of the bioethics service, particularly among those who did not have, or did not access a formal bioethics service. Four themes emerged which described the functioning of a contemporary bioethics service. The themes were: striving for consensus; the importance of guidelines; facilitating a time-sensitive and relevant response; and strong leadership and teamwork.

### Striving for consensus

Many participants saw the bioethics service as an avenue for discussion and an active way to help achieve consensus on the future care pathway of a child, and they felt that discussing treatment options with parents was essential. Several participants described regular debates between clinicians regarding the best form of treatment for a child.

*“… you’ve got one team saying we want to maybe try this, other people saying I’m not sure it’s the right idea, but that’s fine. As long as you can have a respectful dialogue. It would be really bad if we all agreed on everything”* (Participant, United Kingdom).

Without a consensus, participants felt that this could lead to a potential adverse impact on the child and family. Examples of such impacts included having to continue with less-than-optimal treatment, passing on the problem to another institution, and not finding a resolution before a child’s death. In addition, participants acknowledged the personal impact of these ethical decision-making scenarios on the clinicians, and the possibility of moral distress. Several participants discussed the potential for the bioethics service to support staff in such situations, some by providing one-to-one support to help alleviate personal distress: “*burnout, compassion fatigue is a huge problem”* (Participant, South Africa). However, very few reported that this service was part of the functionality of the service in their hospital.

In hospitals where there was no official bioethics service, or the official service was not perceived as useful, some of the participants discussed how they felt the need to develop their own informal or within-unit groups. In some instances, the bioethics services were not perceived as useful to help with discussions around the initiation of I-LTV. Reasons given for this were, for example, that they were designed exclusively for a particular condition, such as paediatric cancer, or, in hospitals where paediatric services were provided alongside adult services, the bioethics services was focused more on adult issues with little understanding or experience in paediatrics. Access was also seen as important for the service to be successful: *“I don’t think we are aware that it is actually there, so they don’t really promote themselves”* (Participant, Denmark).

Many participants described disputes or disagreements with the family as one of the main reasons why the bioethics service was consulted. They saw the role of bioethics as a means of helping everyone understand each other’s concerns, with the goal of reaching consensus on goals of care. However, there was a risk that some families would not trust the hospital bioethics service to be impartial:

*“[the families] can actually have a consult at any point. And we’re happy to coordinate that. …if they don’t believe the clinicians, then they’re not necessarily going to believe the clinician’s colleagues either”* (Participant, United States).

We found that the working structure of the unit impacted the clinicians’ and any bioethics service’s ability to help reach a consensus. One participant, in a clinical role, described how the working rota changed often, resulting in few opportunities for detailed handover of information with colleagues. This contributed to the challenge of building a rapport or creating a bond with the family in a busy clinical environment, and as a result it was difficult to fully understand the child and family’s approach to goals of care:

*“For me this makes any progress difficult because you don’t really have enough time to know the family, explore in depth their views, their preferences … then it’s very difficult to achieve a meaningful decision”* (Participant, United Kingdom).

### The importance of guidelines

All participants work within the legislative framework of their respective countries, and the influence of these will be explored in separate work currently underway as part of the TechChild project. In addition to the legal context, some participants felt that the use of guidelines for bioethics provided a number of benefits, not least in stimulating discussions and allowing different ethical aspects of each discussion to be drawn out and analysed. Following guidance helped to improve objectivity and fairness. For example, one participant stated:

“*I think that’s particularly important you know, for us at least … we’re very white, like many … specialities in medicine. And our patients are [mainly from a] minority [background], specifically black. Here where I am we have a large indigenous population as well. And I think that a standard framework could potentially help to mitigate inputs of bias, or much less explicit bias in who we deem appropriate to receive medical technology.”* (Participant, United States).

Some participants described the different guidelines they used to conduct discussions, both in formal bioethics services and in informal discussions; for example: legal guidance; national clinical guidelines, hospital policy and/or ethical guidelines. Some participants said that reviewing previous cases and discussions helped them develop guidance for future ethical discussions in their institution by identifying bioethical principles, and hospital principles to guide decisions. Hospital policy in particular, seems to have an influence on decision-making and the access or remit of the bioethics service. Many participants felt that it was very difficult for a bioethics service or informal clinician group to argue against hospital policy. For some participants, the consequences of doing so could be extremely challenging, despite their belief that to continue treatment would cause further harm.

*“we don’t have a futility policy in the hospital and so it’s not like to say, no, we’re not going to do it, and again because we would have had all these legal challenges and everything, you know”* (Participant, United States).

Some clinicians felt this to be the case if the hospital tends to prioritise the views of the parents over those of the clinicians. If the clinicians wish to challenge the parents’ treatment decisions, this was either not supported by the institution, or the clinicians were discouraged from objecting to a course of action preferred by the parents. This can be problematic, as one clinician states:

*“if you are not challenging, there won’t be a dispute, but it doesn’t mean that there is no ethical issue”* (Participant, United Kingdom).

In some institutions, the participants stated that they were unaware of any guidelines to follow. Some of the participants stated that clinicians relied on their personal experience of working with children and families to guide their deliberations. Where there were no guidelines available, or no awareness of existing guidance, some participants reflected that they wished there was a more standardised way of considering each child’s situation, or that guidelines existed for such difficult cases:

*“Maybe a guideline could be helpful in guiding decisions with more rationality and less feelings”* (Participant, Italy).

One participant described how their team used basic ethical principles, such as the best interests of the child, do no harm, or distributive justice to guide their discussions and decisions.

*“But whenever we are in a situation where we are not sure of the outcomes of the actual underlying process, we would go back to what would the benefits be for the child*.” (Participant, United Kingdom).

Others discussed how, in the absence of formal guidelines, they try to remove emotions and personal feelings from any discussions and build a picture of the case so they can analyse more objectively. As one participant stated, decisions of such magnitude require “*something more than just personal values and experience*” (Participant, United States). More than one clinician debated out loud if it would be possible to build an algorithm or checklist for guidance in these complex situations, however, there was also recognition that these issues are not so simply solved:

*“it would be wonderful to have some sort of decision tool but the problem is that you are also opening up to people’s feelings and the way they can cope with difficult situations.”* (Participant, Denmark).

Other participants felt that they did not require guidance and were able to manage bioethical discussions and make ethical decisions without assistance:

*“I think we do not follow any guidelines to be honest. I’m quite sure about that. More of a case by case decision and of course it is influenced by the people who are treating the child in that period. It has too many influences”* (Participant, Italy).

Discussions were felt to be very individual, and unique to each situation, and it was felt that those treating the child are best placed to make the decisions without input from bioethics.

### A structure that facilitates a time-sensitive and relevant response

Many participants stressed that in general, using an integrated approach towards discussion allows the child and family to benefit from the wisdom of a team of individuals with different viewpoints. This, however, needs to be balanced against the need for time-sensitive actions. One of the issues raised by participants in the theme of using a formalised process or guidelines for decision-making was that the process would take “*time that we don’t have*” (Participant, United States). Many reported that clinicians could not spend time waiting for a formal service, or for help with guidance to respond to a request for assistance. It was also clear from the interview data that a bioethics service needs to be able to respond appropriately and quickly to the clinicians’ and the parents’ needs. Most of the formal and informal bioethics services attempted to access varied opinions for any deliberations. Many of the participants stated that it was important for a functioning bioethics service to consult hospital clinicians closely connected to the case. It was also deemed essential to consult, where appropriate, with other clinical groups such as palliative care services, nurses, or nursing representatives, religious and cultural leaders, primary care professionals, and social workers, in bioethical discussions. Thus creating “*a pretty eclectic group really*” (Participant, United Kingdom). In addition, a gender balance and a mixture of young and older voices were important.

*“…they have to have law, legal professors or[legal] …representatives, social workers, nurses, physicians. So they all have to have a ratio. And they also have to have … a family or a patient”* (Participant, Taiwan).

Participants discussed the importance of understanding the perspective of families, including their customs and beliefs, which may be quite different to those of the clinicians. This may be represented by lay members in the bioethics discussions, such as religious leaders or other culturally representative individuals. This was sometimes difficult to achieve:

*“I tried to engage with the religious services but the [religious leader] was very difficult to reach and when he finally replied to my messages the patient had been already transferred to another hospitalj”* (Participant, United Kingdom).

The bringing together of all parties created a structure that brings broad experience to a bioethics service, while retaining the ability to address issues in a timely manner is facilitated in different ways. Some participants gave examples of smaller bioethics groups, which gathered and interpreted information about an ethical issue, for a larger group to discuss. Others described services as accessible when they were flexible and quick to respond. Some described a 24-hour on-call service, which responded the same day if the issue was urgent or the next day if it was a more long-term matter. Many participants expressed how a balance must be kept between the need for diversity and the accessibility of the bioethics service. Too large a group may be difficult to convene quickly and too many people in a meeting could be overwhelming, particularly for the parents or family of the child if they are present.

Most participants described how the clinicians only approached the ethics service when they felt it was needed, rather than involving bioethics services in all cases where technology dependence or other potentially ethically challenging circumstances may arise. The majority of participants also stated the service was accessed by means such as using a telephone helpline, electronic medical records, or a dedicated email address to ask for a consultation. Some of these methods were relatively formal, in that the clinician was required to produce a document outlining the case:

*“They’d write a two- or three-page clinical summary describing… the child’s clinical state, …what the diagnosis might or might not be. And putting forward what the proposals might be as alternatives and what the options might be. At which stage then within probably a couple of days the ethics committee would be convened, specifically for this purpose”* (Participant, Ireland).

Others described a more informal or ad hoc process of bioethics consultation, for example, clinicians may ask an individual from the service to visit the unit. The nurses or allied health professionals interviewed expressed that they felt they were more likely to identify a potential dispute than the physicians, or identify earlier if the parents were unhappy or uneasy with the treatment plan and this could be discussed in a smaller bioethics group. In general, the larger bioethics groups met regularly, whether they were formally organised by the institution, or informally meeting with clinicians and others within the PICU. Many participants described their meetings as taking place once a month, or every six weeks; other committees were only convened when an ethical issue was identified as needing their input. Some participants felt that the structure of a bioethics service could make access more difficult. For example, one participant described that they felt the bioethics service was not a safe space for frank discussions, because all submissions had to be made in writing, which prompted a concern that they may be used against the clinicians at a later date.

Some bioethics groups discussed or reviewed cases as part of wider ethical education; and some also provided formal ethics education for clinicians. This was seen as beneficial by the participants and, in some cases, it was felt that education combined with informal bioethics discussions meant that the clinicians could overcome problems within the unit, and did not need to avail themselves of what they saw as an external bioethics service to help them with issues.

In almost all bioethics services or informal groups, the participants stated that training in ethics was regarded as important. Many participants mentioned that one or more members of their bioethics service had qualifications in ethics, such as a Masters’ Degree or PhD training, or they were in the process of training. The qualified individuals were generally the ones who conducted the initial consultation with the clinician(s) and family connected to the bioethical issue, before discussing with the wider clinical team and the wider family where necessary. Some services or institutions require basic training in order to become part of a bioethics service.

### Strong leadership and teamwork

Many of the participants discussed how a successful bioethics service often depended on one strong leader, and the necessity of good teamwork. The strong leader was, in some cases, the participant interviewed as part of the research, the participant’s mentor, or the individual who established and pushed the bioethics service forward. Participants described how crossing the barriers between *“the ethics versus medical fences [which] … was certainly tricky”* (Participant, Ireland). In order to keep the profile of bioethics high, it needs a strong individual to advocate for the clinicians and children undergoing the dilemmas. Particularly in the face of political and cultural changes:

*“When our department Chair was very strong [that person] could just tell everybody no we’re going to be good doctors and that’s what we’re going to do. [Their] successor, who is now gone, didn’t want to be troubled by certain things. And so the hospital and the political shenanigans sort of came more into play”* (Participant, United States).

More than one participant described how colleagues of theirs were part of bioethics services and encouraged ethical thinking around the issues they face from a very early stage. For example, instilling ethical training into practice.

*“And as a group we’ve talked about … in the same way that we have a dedicated chaplain and a dedicated social worker … It would be really nice to have a dedicated ethicist”* (Participant, United States).

One participant described how very few bioethics services, in their experience, were proactive in approaching clinicians about the possible need for bioethical discussions, although this did happen on occasion. Where this had occurred, it was dependent on an individual in the bioethics service who engaged in paediatric issues at an early stage, when it was thought a bioethical issue might arise. Some participants described clinicians who were able to push for bioethics to be discussed sooner in a care pathway, seeing this as a means of generating more understanding and avoiding potential disputes.

*“I think [the individual] pushed our group to think about involving ethics sooner rather than later. … [Otherwise] I might not necessarily think about talking to them on a regular basis”* (Participant, United States).

Conversely, strong individuals also influence clinicians in the opposite direction. A strong leader of a bioethics service may also influence a sub-optimal bioethics response. For example, one participant described how one of the Chairs of a bioethics committee had no formal training in bioethics and also allowed their religious views to influence the discussions. Others described how the absence of a strong leader within a bioethics team, led to the bioethics committee losing confidence in the consulting clinicians, and eventually to the service’s demise. In addition, one participant described how the Head of Department felt strongly that bioethics consultations were of no use, as a result, clinicians did not request a consult in the department, even if they felt bioethics input would be beneficial: *“it’s really hard to challenge them without feeling you are overstepping”* (Participant, United Kingdom). Another described how their superior felt that it would “*create a case*” if the bioethics service was consulted, despite the issue being challenging for the clinicians and the family, effectively preventing bioethical input.

As well as strong leaders, participants described how the bioethics service, whether formal or informal, depended upon good teamwork and communication:

*“they are informal and yes we know each other but because we have these discussions so very often people do not hold back. So even though that you are a nurse or if you are young, you are free to say whatever you actually mean”* (Participant, Denmark).

This varied from descriptions of a team within a ward, working together closely, with good relationships between each other, and good knowledge of other professionals and lay members who can join in with discussions as required; to innovations where ‘inter-city’ teams are established to provide bioethics support over a number of hospitals, or to standardise the basis on which certain treatments will be offered or refused. The creation or the continuation of a bioethics service was described as challenging if there was no underlying functioning team of individuals. One participant described how, when faced with navigating on-call rotas, and availability of potential members, it became almost impossible to establish a functioning bioethics service. Some participants described how they had attempted to establish bioethics services in their own institutions or across their region in order to support clinicians and parents in decision-making. Although they had been locally successful, the continued inconsistency of remit means that the support from bioethics services varies depending on local institutional support and reputation.

*“they discussed setting [the paediatric clinical ethics service] up … they wanted to … establish the adult service before doing a paediatrics one. And then see what the throughput was like.”* (Participant, United Kingdom).

Sometimes the need for a regional approach is stimulated by challenging cases. One participant described how, on occasion, families would visit several hospitals in their region, asking for treatment that had been judged as inappropriate by clinicians. The bioethics service in several of these institutions aimed to achieve a joint consensus in their approach to such issues, ensuring all viewpoints were considered.

In the absence of a structure that allows the development of a closely communicating team on the unit, several participants mentioned incorporating the presence of another treating team as a good way of including ethical thought, and of being beneficial to the treating clinicians in providing a different view. Several participants mentioned the support, as clinicians, they received once a palliative care team became involved in discussions. Some of these participants were also members of a palliative care team, as well as being involved in bioethics services. The input of the palliative team was seen as able to provide a more objective perspective and build trust and communication with the family. Such teams were seen as providing a sense of continuity for the patients, where in the clinical teams this is not always possible when the PICU clinicians’ rota changes every week, and they do not get to know the patients and their concerns in as much depth.

*“I think that there is… implicit bias and explicit bias that we have as clinicians. I think that what we try to do as a palliative care team is to kind of zoom out when we’re talking to the team and remind the team that this is the family’s first time navigating this and these decisions, and while we as providers have navigated this and walked this journey with many families, we know the process, we know the outcome.”* (Participant, United States).

## Discussion

Bioethics as a service is increasingly called upon to address novel medical ethical issues, and clinicians are required to consider and make decisions on ethical challenges that previously did not exist [1, 11, 21, 22]. The iterative nature of qualitative research undertaken as part of the TechChild programme identified the functionality of bioethics as an important factor. During the analysis of these data, and in discussion of the themes, our findings strongly resonated with the well-established Rogers’ Theory of Diffusion of Innovation [15] particularly in the exploration of the implications of our findings. This theory was useful to interpret our findings against and inform how the bioethics services may be developed in the future.

The themes we identified speak to the functionality and structure of bioethics services. In addition, they create a rich description of the issues that clinical bioethics services address, or fail to address, within the dynamic environment of ever-sophisticated life-sustaining technology used in PICU. The first two themes describe the purpose of bioethics services, outlining the need for them, and the remit that they need to have if they are to work successfully, the need for consensus before a decision is made, and the guidelines or principles that a bioethics service can follow. The remaining two themes describe how bioethics services achieve, or are prevented from achieving, their purpose, through their structure and the drive of the individuals that are involved.

### Issues of consensus

Effective and considerate communication between clinicians, patients, and their families is an important facet of modern medicine [20–25]. This is particularly pertinent at a point of care when a child’s survival is dependent on the treatment available or offered, the child’s condition is complex, and the limits of what is clinically possible are being tested. It has previously been identified that skills in communication and trust to identify the goals of care and treatment plans are of the utmost importance in contemporary medicine [1, 2, 20, 24–26]. Much of the conversation in our participant interviews about the role of bioethics emerged from the need for consensus between the clinical team and the parents and family of the child. We found that bioethics services were often described as being availed of if there was a dispute, or if there was the potential for dispute. The risk of dispute or the consequences of dispute has been recognised as one of the major stressors when technology dependence is initiated [1, 2, 5, 21, 22, 24], and can even become the focus of widespread attention, involving the legal system and wider media coverage [21, 27, 28]. Some participants saw the potential of a bioethics service to help reduce this risk, particularly if bioethics were involved in discussions from an early stage, before a dispute can arise; potential that is reflected in the scientific literature [24, 29]. A focus on creating a culture which embraces bioethical support, and normalises accessing the service at an early stage, as opposed to short-notice when conflict has arisen, may well be a productive way forward for the functionality of bioethics services.

Changing attitudes towards technology, and greater access to information about the potentials of new technology change the nature of how consensus can be achieved. In previous work conducted by the TechChild team we found that clinicians sometimes felt frustrated trying to explain why a particular technology may be appropriate, or inappropriate, for an individual child [22]. In addition, cultural attitudes towards medical advancements have changed, for example Glover-Thomas [7] described how sometimes death was seen as an insurmountable obstacle. These issues, among others, seem to be part of the grounding of bioethical consideration within changing cultural norms. The bioethics services therefore increasingly see their function as important where issues are debated and clarified, and individuals can be supported in their own wellbeing [6, 21, 30].

### Use of guidelines

One of the principal uses of guidelines is to ensure justice and fairness in decision-making as well as providing some consistency, which may take the burden from decision-makers where there is a challenging issue to resolve [21, 31, 32]. The discussions about guidelines described the types of guidelines that were used, and the influence these had on deliberations; using no guidelines but working from individual or team experiences and values; and using previous cases and experiences to develop their own guidelines to help guide ethically challenging situations. Our finding of the inconsistency of guideline usage to some extent reflects how guidance is generally successful when it is relevant to the social and cultural norms of the environment in which those in bioethics services work [33–35]. The choice not to use guidelines may reflect an inadequacy of socialisation of guidance, particularly around technology dependence, where possibilities to sustain life are increasing. Where a team uses only their own judgement based on previous experience and personal values, this may reflect more accurately local norms and customs. However, there is a much greater risk of implicit bias influencing decisions. Examples of implicit bias in the discussion of life-sustaining technology or complex treatments have been discussed in the literature, particularly in the context of using guidelines or decision strategies to try to avoid potential bias [36–38]. Crosskerry [37] described the many different biases that occur in reasoning, some of which are reflected in our findings. Clinicians may discuss with individuals who have similar views or experiences and be drawn to those who are likely to agree with their point of view.

An unintended consequence of any bias may contribute to misunderstanding and dispute where values differ between parties. Implicit bias has been described as a factor in reinforcing uneven power balances in clinical bioethical discussions [39], which we recognise as some of our participants discussed ‘persuading’ parents to a particular point of view. Arguably, one of the functions of a bioethics service is to increase transparency about the influences on these discussions, by using guidelines or making explicit the viewpoints discussed.

### Structure that facilitates a time-sensitive and relevant response

An important element of the functionality of the bioethics service is its structure. This is described in terms of the individuals who make up the service, how the service is accessed and how it functions. Scientific literature underlines the importance of a balance of different views and the value of a collective approach by bioethical services [1, 2]. Our interviews showed a broad understanding of this concept, but also the need for a timely and less intimidating service, as has been previously discussed [1, 2, 5], which has been shown not to compromise any effect of bias [36]. Training in order to contribute to bioethics services, and in some cases qualifications, are known to be important [2, 6], and are a factor recognised in our interviews as well as the role of bioethics in education. However, the answer to time-sensitive discussions is in plain sight, in the form of increasing the input of palliative care teams [40] as participants highlighted the value of continuity of care provided by the palliative care team when included earlier in a patients’ journey, in terms of providing experienced ethical thought, building trust and communication with the family.

### Strong leadership and teamwork

Several participants mentioned the presence of a strong leader, or a passionate individual who was key in creating or driving the work of a bioethics service in their institution, or conversely, the lack of such an individual meant that the bioethics service did not continue or was not used. Ideally a robust bioethics service, and a culture which embraces it, should not be reliant on the strong personality of one person. However, a strong leader who is a positive role model for seeking ethical advice will help to cultivate this approach within their teams. Unfortunately, as evidenced by our findings, the latter is also true and could mean that some clinicians are desperately seeking ethical guidance but are hindered by hierarchy and strong personalities. The literature on innovations and organisations [23, 41] describes the benefits, and weaknesses of relying on an individual to push innovations forward. The chair of a bioethics meeting is required to demonstrate strong leadership qualities to ensure all participants are heard, and that decisions are not compromised by institutional norms or biases [39].

Another factor that emerged from the interviews is the reliance on collaborative teamwork to achieve consensus and provide support for the parents and clinicians facing an ethical dilemma. Research has shown that effective teams communicate with trust and meet regularly; and this increases confidence in a bioethics service [6, 22, 23]. This was certainly evidenced in our research. Conversely, in institutions where teamwork is prevented by staff turnover, shift patterns or poor communication, the use of bioethics services, or the ability to create an informal bioethics discussion group in the absence of a functioning clinical bioethics service, is severely compromised [2].

### Assessing the functionality of a bioethics service

The thematic analysis provides important information about what constitutes a successfully functioning bioethics service, but it is evident that this is a service that is not consistently adopted or used at a time when technology is rapidly changing, and the need for ethical advice is arguably more acute than ever before [14]. Mapping our findings against the well-established Rogers’ Theory of Diffusion of Innovation [15] helps understand how, if we regard contemporary clinical bioethics as an innovation, there is potential for relevant bioethical support that can be adopted by different institutions in different cultural contexts. Rogers’ Theory describes how an innovation is communicated through certain channels over time among members of a social system. The four main elements relevant to this research can be seen as (1) The concept of clinical bioethics service as an innovation; (2) The power of communication in the adoption of the innovation; (3) The time needed to facilitate adoption of the clinical bioethics service; and (4) The social system surrounding bioethics services.

**Fig. 1:**
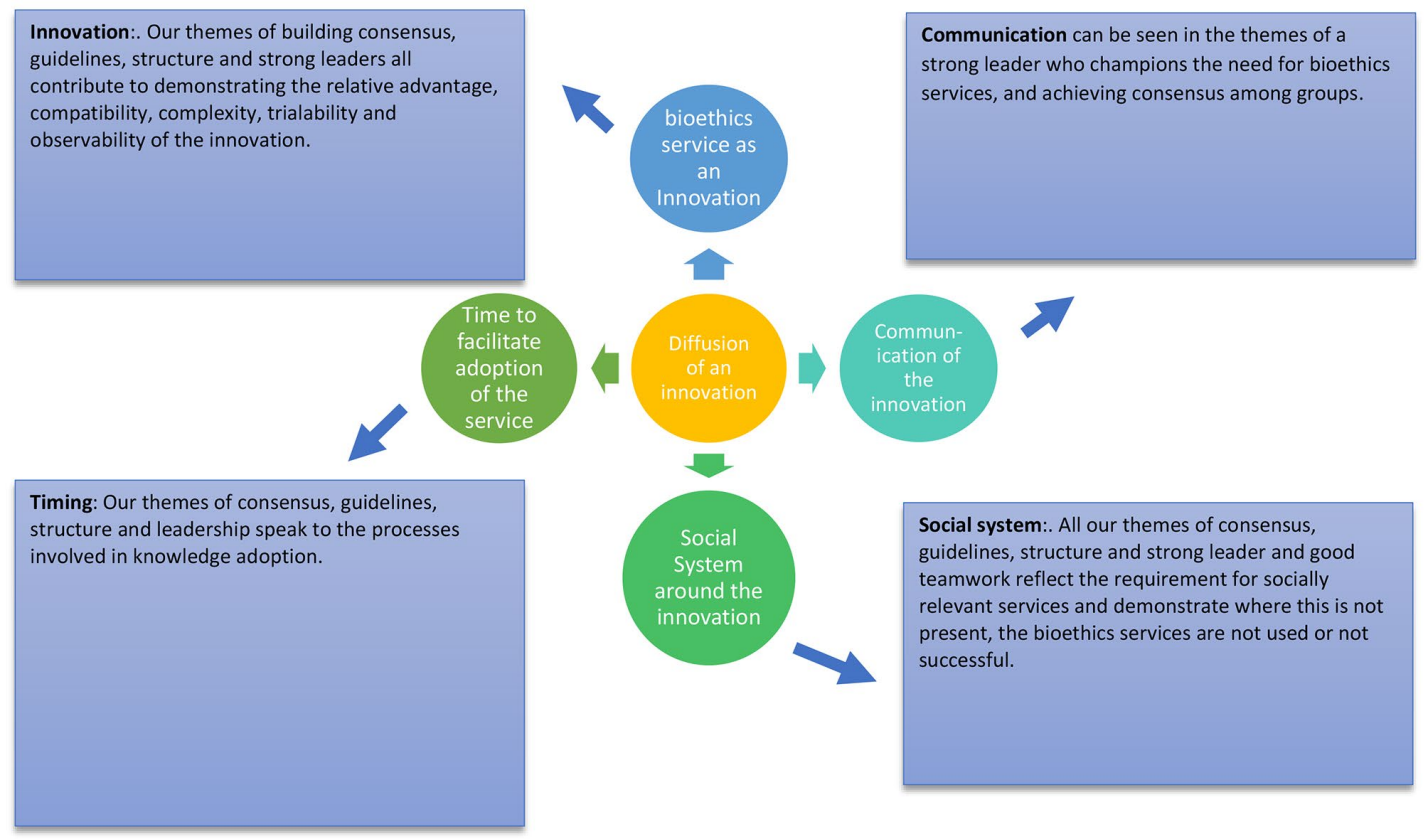
Themes mapped against Rogers’ Theory of Diffusion of Innovation [15]

Figure 1 demonstrates the key elements of Rogers’ theory of Diffusion of Innovation [15] in terms of the identified themes in our results. Our results show examples within the themes of the need to find a consensus, the benefits of a wide structure, use of guidelines and the need for an effective leader who supports the use of and functioning of the bioethics service. Figure 1 maps the findings against the Theory of Diffusion of Innovation [15].

### Limitations

The international and multi-site design of the study allowed a diverse and varied representation of views. Results found variations in terms of the structure and functioning of bioethics services. However, the variation and inconsistency found within the findings are reflective of the current lack of contemporary guidelines that guide clinicians in the hospital setting. Interviews were conducted with participants based in countries that all had diverse cultural, social and legal values that significantly influence the structure and operation of bioethics services. There may also be an element of selection bias to this study, as participants were able to take part in the study based on their expressed interest in the topic. However, the findings have provided deep and valuable insights into the functioning of clinical bioethics services from the perspectives of clinicians who are directly involved.

## Conclusions

Considerable challenges are faced by clinicians and other decision-makers when addressing bioethical issues at the point of care when a child requires technology assistance to sustain life. The influence of bias (implicit and explicit) or personal value judgements may lead to dispute at a critical point of care delivery. Bioethics services have an important role in clarifying conflict and assisting with navigating dilemmas, which may secondarily reduce stress, but they are not, at present, perceived as functioning optimally in all contexts in order to achieve this aim. Our rich description of the experience of bioethics services from the perspectives of physicians, nurses and those in allied health professionals has found that it is often perceived as not being a service that is fit-for-purpose to deal with contemporary and often time-sensitive issues that arise. When mapped against Rogers’ Theory of Innovation, the findings demonstrate attempts to improve discussion and consensus, the use of guidelines to reduce bias, a broad structure to reflect different values and goals of care and often the presence of strong leadership and teamwork, and how these factors can contribute to a more innovative approach to bioethics services. At present it is evident that the full potential of bioethics services are often not being realised. There is therefore a need for access to a time-sensitive flexible bioethics service for all PICUs which is underpinned by ethical, legal and bioethical expertise. It is critical that this service has a strong inclusive leadership. In parallel there is a need for increased inclusion of palliative care teams, as early as possible on a child’s journey, to support trust in difficult decisions through continuity of care.

### Electronic supplementary material

Below is the link to the electronic supplementary material.

Supplementary Material 1

## Data Availability

The datasets generated and/or analysed during the current study are not publicly available due to the risk of breach of confidentiality, including the possible identification of participants or of cases discussed in the interviews. Pseudo-anonymised transcript data are available from the corresponding author on reasonable request.

## List of abbreviations

ECHO: European Children’s Hospital Organisation
ERC: European Research Council
GDPR: General Data Protection Regulation
I-LTV: Invasive Long-Term Ventilation
PICU: Paediatric Intensive Care Unit
PIL: Participant Information Leaflet
VPN: Virtual Private Network
